# High prevalence of coronavirus NL63 in SARS-CoV-2 negative COVID-19 suspected patients

**DOI:** 10.1016/j.nmni.2023.101159

**Published:** 2023-06-08

**Authors:** Roya Sadidi, Amir Azimian

**Affiliations:** Department of Biology, College of Basic Sciences, Damghan Branch, Islamic Azad University, Damgan, Iran; Department of Pathobiology and Laboratory Sciences, Faculty of Medicine, North Khorasan University of Medical Sciences, Bojnurd, Iran

Dear Editor,

In the last months of 2019, an increasing number of cases of pneumonia were reported in Wuhan, China. The etiology of this disease was unknown, and most affected patients had complicated respiratory symptoms related to respiratory illness, including cough, fever, headache, and breathlessness. At the same time, some showed respiratory failure, acute respiratory distress syndrome, shock, and death. More evaluation of dead patient samples and genome sequencing led to confirming a novel coronavirus, is named SARS-CoV-2 [1]. This virus is highly transmissible and, to date reported in most parts of the world, including Iran. To date, more than 680 million cases of disease and more than 6.8 million cases of death have been reported in the world, and near to 7.6 million cases of disease and more than 144000 cases of death have been reported in Iran [1]. From the beginning of the pandemic to date, we had seven waves of the disease in Iran. The first wave was in the spring of 2020, the second was in the last months of the summer, and the third wave was in the autumn. After the seventh wave in the last months of 2022, the incidence of covid-19 decreased significantly in most parts of Iran. However, we had many patients with symptoms of covid-19 while the rt-PCR test was negative. By further evaluating of the patient's record, we found that the patients' symptoms were similar to those of viral pneumonia rather than bacterial. So, we decided to examine the presence of some important respiratory pathogenic viruses, including influenza A virus, Coronaviridae (229E, HKU1, NL63, OC43), rhinoviruses, metapneumovirus, bocavirus, RSV, and parainfluenza virus [1,2] in the same SARS-CoV-2 negative samples of the SARS-CoV-2 negative covid-19 suspected patients to find the reason of this signs and symptoms in the covid-19 negative patients. The samples were selected from patients admitted between June 2022 to 2023 January in the referral hospitals of North Khorasan province, Iran.

## 1 Material and methods

We evaluated 600 SARS-CoV-2 negative covid-19 suspected samples, between 2022 June to 2023 January in the referral hospitals of North Khorasan province, Iran. Most of them suffered from the acute respiratory syndrome. Clinical presentation, radiological features, and laboratory findings were collected from electronic medical records. We evaluated selected samples to detect some important respiratory viruses, including the influenza A virus, Coronaviridae (229E, HKU1, NL63, OC43), rhinoviruses, metapneumovirus, bocavirus, RSV, and parainfluenza virus. The nasopharyngeal and throat swabs of selected patients were evaluated for COVID-19 using the addbio RNA extraction kit (ADD BIO Inc, Daejeon, Republic of Korea) and LightMix Modular SARS-CoV-2 probe and primers (TIB molbiol, Berlin, Germany) and addbio one-step RT master mix (ADD BIO Inc, Daejeon, Republic of Korea). The other respiratory viruses were evaluated using previously described methods [1,2]. We also got other related information such as laboratory tests and chest computed tomography scan results, and physical examination data from the patients' files with the license of the ethics committee of North Khorasan University of Medical Sciences (No.: 980002).

## 2 Results

We evaluated 600 SARS-Cov-2 negative samples of suspected covid-19 patients between June 2022 to 2023 January in the referral hospitals of North Khorasan province, Iran. Of these, 409 cases were negative, and 191 were positive for evaluated viruses. 126 out of 191 positive tests were NL63 coronavirus, and 28 samples were positive for *influenza A virus*. We also had positive samples for *OC43 coronavirus*, *Rhinoviruses*, *Metapneumovirus*, *Bocavirus,* and *Respiratory Syncytial Virus* [Table 1]. All *Rhinovirus* and *Metapneumovirus* positive cases belonged to 0–14 years old patients, and also all OC63 *Coronavirus,* and *RSV* isolates were isolated from more than 60 years old patients, and *Bocavirus* just found in the 46–60 years old group. NL63 *Coronavirus* was seen in all age ranges, and bilateral pneumonia was the most frequent finding in CT scan evaluation. 229E and HKU1 Coronaviruses, Bocavirus and RSV didn't have any CT scan findings. It should be noted that Ground glass opacity was just seen in NL63-positive patients.

**Table 1:** Frequency of other respiratory viruses and clinical signs in Covid-19 negative cases.

		Age (n/%)				Clinical signs (n/%)								
		0–14	14–40	40–60	>60	CT scan findings			Diarrhea	Headache	Fever	Cough	Sore throat	Fatigue
						Unilateral pneumoniae	Bilateral pneumoniae	Ground glass opacity						
Influenza A		–	7 (25%)	12 (42.9%)	9 (32.1%)	12 (42.9%)	16 (57.1%)	–	18 (64.3%)	28 (100%)	28 (100%)	28 (100%)	19	28
Coronavirus	229E	–	–	–	–	–	–	–	–	–	–	–	–	–
	HKU1	–	–	–	–	–	–	–	–	–	–	–	–	–
	NL63	36 (28.6%)	12 (9.5%)	30 (23.8%)	48 (38.1%)	18 (14.3%)	60 (47.6%)	48 (38.1%)	78 (61.9%)	120 (95.2%)	108 (85.7%)	120 (95.2%)	114	120
	OC43	–	–	–	2 (100%)	2 (100%)	–	–	–	2 (100%)	2 (100%)	2 (100%)	2 (100%)	2 (100%)
Rhinoviruses		14 (100%)	–	–	–	6 (42.8%)	–	–	–	7 (50%)	–	14 (100%)	13 (92.8%)	6 (42.8%)
Metapneumovirus		8 (100%)	–	–	–	8 (100%)	–	–	–	8 (100%)	8 (100%)	8 (100%)	8 (100%)	–
Bocavirus		–	–	6 (100%)	–	–	–	–	6 (100%)	–	–	6 (100%)	6 (100%)	–
Respiratory Syncytial Virus		–	–	–	7 (100%)	–	–	–	–	7 (100%)	–	7 (100%)	7 (100%)	–
Parainfluenza Virus	1	–	–		–	–	–	–	–	–	–	–	–	–
	2	–	–	–	–	–	–	–	–	–	–	–	–	–
	3	–	–	–	–	–	–	–	–	–	–	–	–	–
	4	–	–	–	–	–	–	–	–	–	–	–	–	–

## 3 Discussion

To date, we have increasing reports of SARS-CoV-2 negative COVID-19 suspected patients. In addition, we have SARS-CoV-2-positive patients without response to COVID-19 treatments. In the first cases, we should evaluate the other respiratory viral and bacterial infections, and in the second cases, we should evaluate the Co-infections. Regarding Co-infections, many papers have been published during the COVID-19 era [1,3]. Most reported Co-infection with the *Influenza A* virus [3]. Co-infection with other respiratory viruses such as *RSV*, *Metapneumovirus*, *Bocavirus* and *Adenovirus*, and bacteria and fungi is also reported in many reports [1,3]. In this research, we worked on respiratory viruses in SARS-CoV-2 COVID-19 suspected patients. Interestingly, we found a very high prevalence (66%) of NL63 *Coronavirus* in these patients. 14.7% were *Influenza A* positive, and low numbers of other respiratory viruses, including OC43 coronavirus (1%), Rhinovirus (7.3%), Metapneumovirus (4.2%), Bocavirus (3.1%), and RSV (3.7%). In some previous papers, researchers mentioned a similar mechanism of cell entrance for *coronavirus* NL63 and SARS-CoV. They found both viruses use the ACE-2 receptor [1,4]. One of the reasons for the decline in Covid-19 positives in recent days may be the winning of *coronavirus* NL63 over SARS-CoV-2 in connection with the ACE-2 receptors and more NL63 *Coronavirus* in the environment in comparison to SARS-CoV-2. The similarity between the S protein of seasonal Coronaviruses and SARS-CoV-2 may look good at first vision. However, there are many reports proved previous infection with seasonal Coronaviruses not only not helpful for the human body, but also harmful cross-reactions may lead to severe immune responses to SARS-CoV-2 in the following exposures [4,5]. These are primary hypothesis and needs to be thoroughly evaluated in various aspects. To prove the role of this virus, more samples should be evaluated, and other intervention factors should be eliminated.

## Funding source

This work is part of the national COVID-19 screening program of Iran. The Primer and Probes were supported by the 10.13039/100009647Ministry of Health of Iran, and other laboratory kits were provided by the North Khorasan University of Medical Sciences [grant number 990002].

## Financial disclosure

The authors have no financial relationships relevant to this article to disclose.

## Declaration of competing interest

The authors declare that they have no known competing financial interests or personal relationships that could have appeared to influence the work reported in this paper.
